# Absolute Molar Mass Determination in Mixed Solvents. 3. Accuracy of ∂*n*/∂*c* Values Obtained by Assuming 100% SEC Mass Recovery

**DOI:** 10.1007/s10337-025-04438-z

**Published:** 2025-10-17

**Authors:** André M. Striegel

**Affiliations:** 1 Chemical Sciences Division, National Institute of Standards and Technology (NIST), 100 Bureau Drive, MS 8390, Gaithersburg, MD 20899-8390, USA

**Keywords:** Size-exclusion chromatography, Specific refractive index increment, 100% mass recovery method, Absolute molar mass, Preferential solvation

## Abstract

Accurate values of the specific refractive index increment (∂*n*/∂*c*) are essential to the accurate determination of molar mass averages, distributions, and related macromolecular parameters by, among others, size-based separations with online static light scattering and differential refractive index (DRI) detection. Examined here is the 100% mass recovery method of calculating the ∂*n*/∂*c* of dilute macromolecular solutions, when employing mixed solvents in a size-based separation (e.g., size-exclusion chromatography or SEC) with DRI detection. This method has been used successfully in the past for determining the ∂*n*/∂*c* of various polyelectrolytes. It is quicker, generally simpler, and less sample-intensive than its offline, batch-mode DRI counterpart. Whether or not the 100% mass recovery method allows for the necessary solvent equilibration within the immediate vicinity of the polymer chain during a chromatographic run, so as to allow for accurate determination of ∂*n*/∂*c* in SEC/DRI experiments, is evaluated here. This is done using a set of three narrow-dispersity linear polystyrene standards covering a 40-fold range in molar mass, dissolved in a 25:75 mix of tetrahydrofuran and *N*,*N*-dimethyl formamide. Results are compared to those previously obtained by the batch-mode method and from calculations involving the accurately known molar masses of the polymers and refractive indices of the solvents. The 100% mass recovery method of obtaining ∂*n*/∂*c* values, while of great help for determining the ∂*n*/∂*c* of, e.g., polyelectrolytes, does not appear able to overcome the obstacle of preferential solvation when analyzing macromolecules in a mix of non-isorefractive solvents with dissimilar second virial coefficients.

## Introduction

Accurate determination of macromolecular molar mass (*M*) averages and distributions is crucial to evaluating novel synthetic procedures, as well as to optimizing the processing and end-use properties of materials [1]. Most *M* determinations are performed using size-based separation methods, most commonly size-exclusion chromatography (SEC) but also hydrodynamic chromatography (HDC) and flow field-flow fractionation (flow FFF) techniques [2–4]. In combination with these, the most accurate means of determining *M* is generally through the use of online static light scattering detection, most commonly multi-angle static light scattering (MALS) in combination with concentration-sensitive detection such as differential refractometry (DRI) [1, 5]. A crucial value in static light scattering and DRI calculations is the specific refractive index increment (∂*n*/∂*c*) of the analyte solution. This value corresponds to the change in the refractive index of a dilute analyte solution as a function of analyte concentration, at a given solvent, temperature, and wavelength of incident light. The accuracy with which the ∂*n*/∂*c* is known directly translates into the accuracy of calculated *M* and related values. As such, an error of *E* % in ∂*n*/∂*c* will translate into an error of *E* % in weight-average molar mass (*M*_w_), when the latter is determined by SEC/MALS/DRI. Additionally, most size-based experiments are conducted in neat solvents or in solvents with only a modest amount of additive (e.g., salts). While the use of mixed solvents is common in interaction polymer chromatography methods (e.g., gradient polymer elution chromatography [6]), which have traditionally employed evaporative mass detectors, this is not the case in size-based methods [7]. The latter is due to the widespread use of differential detectors in size-based methods and to the propensity for preferential solvation of macromolecules in mixed solvents. Preferential solvation refers to the greater “affinity” of the macromolecule toward one solvent over the other in a mix, as initially demonstrated by the classic work of H. Kneebone Tompkins in 1896 [8]. When employing differential detection methods such as MALS or DRI, preferential solvation translates into the solvent baseline no longer accurately representing the solvent contribution to the peak height of chromatographic (or fractographic, henceforth implied) slices.

Recent work toward the determination of accurate *M* averages and distributions by SEC/MALS/DRI in mixed solvents has shown that it is possible to obtain accurate values under certain conditions. These conditions include the use of an isorefractive solvent pair, and also, the use of a near-isovirial solvent pair, i.e., when using solvents which, at the experimental conditions, possess either identical refractive indices and/or which allow for dilute polymer solutions with similar second virial coefficients. (The second virial coefficient serves to provide a quantitative measure of analyte solvation at a particular set of solvent/temperature conditions). Details, requirements, and caveats in each case can be found in references [9, 10]. Also examined in reference [10] was the amount of preferential solvation undergone by polystyrene (PS) in a 25:75 mix of tetrahydrofuran (THF) and *N*,*N*-dimethyl formamide (DMF), two solvents which are neither isorefractive nor near-isovirial. Employing polystyrene standards with accurately determined *M* averages and distributions and ∂*n*/∂*c* values obtained by offline, batch-mode DRI (otherwise considered a benchmark method of determining ∂*n*/∂*c*), it was shown that these ∂*n*/∂*c* values were biased due to preferential solvation. Moreover, it was shown that in the 25:75 THF:DMF mix, the solvent ratio in the immediate vicinity of the macromolecules examined was actually 75:25 THF:DMF, due to the greater affinity of PS toward THF over DMF. This resulted in errors in calculated *M*, of up to 1 × 10^5^ g mol^−1^ for PS with *M* = 8 × 10^5^ g mol^−1^.

Another method for determining the ∂*n*/∂*c* of a dilute macromolecular solution at a particular set of experimental conditions (wavelength, temperature, and solvent composition) is to assume that the concentration of the solution has been determined accurately and that 100% of the injected polymer has eluted from the SEC column (or appropriate separation device) and is contained within the integration limits and baseline of the DRI peak (it is also assumed that the calibration constant of the DRI detector is accurately known). This is generally referred to as the “100% mass recovery method” of determining ∂*n*/∂*c*, or sometimes as the “∂*n*/∂*c* from peak method” [11] and has been employed to determine this parameter for dilute polyelectrolyte solutions. When studying polyelectrolytes, it is often assumed that the chromatographic column acts as a type of dialysis chamber so that, over the course of thousands of theoretical plates, equilibration occurs within the solvated region of the macromolecule [12]. The method is generally quicker, less sample-intensive, and easier to perform than its offline, batch-mode DRI counterpart and has found to yield, in a great many cases, ∂*n*/∂*c* values almost identical to those obtained by offline, batch-mode DRI with sample dialysis and other appropriate procedures [12–15]. Given the method’s success for polyelectrolytes, it can naturally be asked whether the same type of equilibration during a chromatographic run occurs when analyzing a neutral macromolecule in a mix of two solvents so that, at the end of the run, the effects of preferential solvation are obviated within the immediate vicinity of the polymer chain. Because the accuracy of the 100% mass recovery option was not evaluated in [10], it is done here, for the three narrow-dispersity linear PS standards previously analyzed, in a 25:75 THF:DMF mix and covering a 40-fold range in *M*.

## Experimental

The SEC/MALS/DRI; absolute refractive index (aRI); and offline, batch-mode DRI ∂*n*/∂*c* methods; along with the PS standards and the solvents employed; have all been described in detail in [10]. As such, only the 100% mass recovery method is described here.

The determination of ∂*n*/∂*c* by the 100% mass recovery method employed the same experimental runs as did the SEC/MALS/DRI determinations of the *M* averages and molar mass distributions (MMDs) of the PS standards. For all analyses, concentrations were well below the critical overlap concentration c*. The calibration constant of the DRI was determined by the instrument manufacturer using a series of NaCl solutions, the ∂*n*/∂*c* of which is well known (0.1740 mL g^−1^ in water at 25 °C at a vacuum wavelength *λ*_0_ of 658 nm). The aRI of the 25:75 THF:DMF mix was 1.4224, measured using the aRI capability of the same DRI as employed in the SEC experiments, at the same experimental conditions (*λ*_0_ = 658 nm, temperature = (25.0 ± 0.1) °C). At these conditions, the ∂*n*/∂*c* of PS as determined by offline, batch-mode DRI experiments was (0.168 ± 0.001) mL^−1^ g (note a typo in Table 1 of reference [10], where the units of ∂*n*/∂*c* are incorrectly stated in the table footnote as being mg mL^−1^).

All data acquisition and processing were performed using Astra software (Waters/Wyatt, version 7.3.2.21).

## Results and Discussion

Three narrow-dispersity (*Ð* ≤ 1.01), linear PS standards were examined, with nominal molar masses of 1.8 × 10^4^ g mol^−1^, 4.20 × 10^5^ g mol^−1^, and 8.00 × 10^5^ g mol^−1^; exact values of the various *M* averages are given for each standard in [10], as are their MMDs. As was shown in [10], for the SEC/MALS/DRI analysis of each PS, the ∂*n*/∂*c* value which provides accurate *M* values in 25:75 THF:DMF at 25 °C and 658 nm is (0.185 ± 0.001) mL g^−1^. This value was consistent across all runs of all three standards (each standard was analyzed at least in triplicate) and was arrived at by incrementally changing the ∂*n*/∂*c* in the Astra software until a value was found which yielded *M* averages in agreement, to within ± 100 g mol^−1^, with those obtained in either neat THF or neat toluene (as has been shown, both the latter values coincided with each other remarkably well).

The above, calculated, ∂*n*/∂*c* value of 0.185 mL g^−1^ differs markedly from the value of 0.168 mL g^−1^ determined from offline, batch-mode DRI, i.e., by injecting solutions of carefully measured concentration directly into the DRI, with the DRI decoupled from the SEC column. The large difference between these two values is due to the preferential solvation of PS by THF over DMF, resulting in a “flipping” of the solvent ratios, from 25:75 THF:DMF away from the polymer to 75:25 THF:DMF in its immediate vicinity. The lower ∂*n*/∂*c* value of 0.168 mL g^−1^ is due to the higher refractive index of DMF vis-à-vis THF, combined with the fact that the solvent injections used to establish a baseline solvent value in the offline, batch-mode experiments are composed of 25:75 THF:DMF. However, when the individual PS solutions of different concentrations are injected directly into the DRI, preferential solvation has changed this ratio in the vicinity of the macromolecules, invalidating the accuracy of the solvent baselines as representative of the true solvent contribution to each injection plateau.

**Fig. 1:**
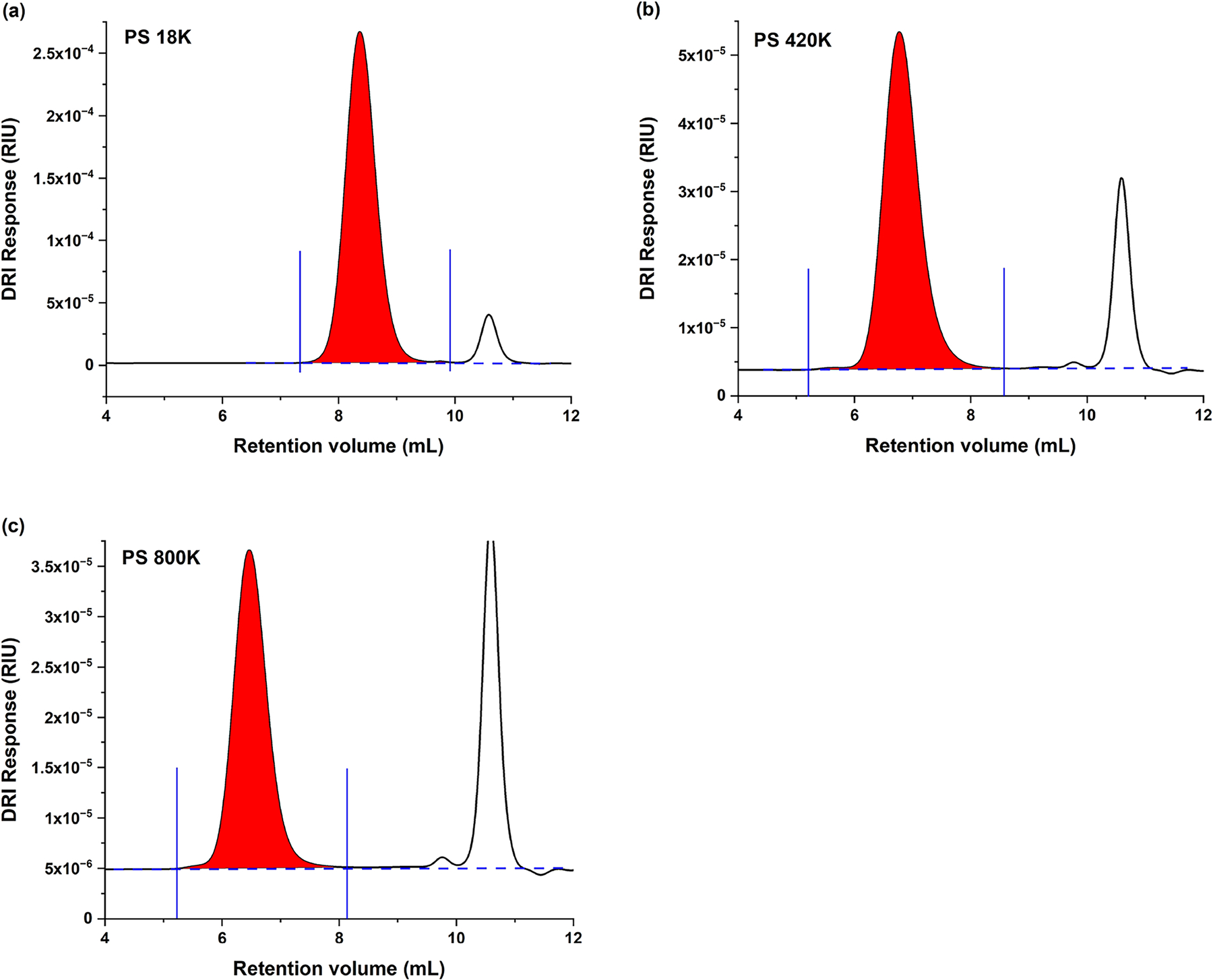
SEC/DRI chromatograms of **a** PS 18K, **b** PS 420K, and **c** PS 800K, in 25:75 THF:DMF, as used for the determination of ∂*n*/∂*c* by 100% mass recovery method. Baseline is denoted by horizontal dashed blue line, peak integration limits by vertical solid blue lines; integrated peak is shown in red. Note that, due to preferential solvation, the baseline does not accurately represent the solvent contribution to the height of each chromatographic slice. Differences in relative heights between analyte (red peak) and solvent/air peak (peak eluting at 10.6 mL) across chromatograms are due to differences in sample concentration, 10 mg mL^−1^ for PS 18K, 2 mg mL^−1^ for PS 420K, and 1 mg mL^−1^ for PS 800K. See [10] for experimental details

Employing SEC/DRI peaks such as those shown in Fig. 1, the ∂*n*/∂*c* was calculated for each PS standard by the 100% mass recovery method. The results are given in Table 1.

The ∂*n*/∂*c* results from Table 1 agree remarkably well with those from offline, batch-mode DRI under the same conditions, i.e., 0.163–0.164 mL g^−1^ by the 100% mass recovery method, 0.168 mL g^−1^ by offline, batch-mode DRI. This coincidence seems a clear indication that in the SEC/DRI case, just as in the offline, batch-mode DRI case, the solvent baseline does not accurately represent the contribution of the mixed solvent to the height of each chromatographic slice. Moreover, it seems clear that passage through the SEC column has not appreciably changed, if it has changed at all, the level of preferential solvation of the PS standards, regardless of *M*. Results were independent of chromatographic flow rate, as the same results were obtained at 1.0 mL min^−1^, 0.5 mL min^−1^, and 0.25 mL min^−1^. The results also appear independent of solvation time, as the lower-*M* PS 18K was analyzed both just a few hours after it appeared to dissolve and after dissolving/solvating overnight; the higher-*M* PS 420K and PS 800K were only analyzed after overnight dissolution/solvation.

The consequence of the above is that, for the PS standards analyzed in a 25:75 THF:DMF mix, *M* averages and distributions calculated using ∂*n*/∂*c* values obtained using the 100% mass recovery method will be in error by almost the same amount as when using ∂*n*/∂*c* values obtained from offline, batch-mode DRI experiments.

It should be noted in closing that the determination of ∂*n*/∂*c* by the 100% mass recovery method is accurate when employing an isorefractive and/or a nearly-isovirial solvent mix, i.e., a mix of two solvents with the same refractive index as each other at the experimental conditions or a mix of two solvents in which the polymer solution has nearly-equal second virial coefficients. In such cases, the effects of preferential solvation on ∂*n*/∂*c* are nullified, as has been shown in [9, 10]. Indeed, when reprocessing the SEC/DRI data from [9, 10] using the 100% mass recovery method, the ∂*n*/∂*c* values thus calculated were found to be statistically identical to the offline, batch-mode DRI-determined values given in those publications.

## Conclusions

The 100% mass recovery method of obtaining specific refractive index increment values, while of great help for determining the ∂*n*/∂*c* of, e.g., polyelectrolytes, does not appear able to overcome the obstacle of preferential solvation when analyzing macromolecules in a mix of non-isorefractive solvents with dissimilar second virial coefficients. This has been shown here for a series of three narrow-dispersity linear PS standards in a 25:75 THF:DMF mix and covering a 40-fold range in molar mass. For these analytes, the ∂*n*/∂*c* values of 0.163–0.164 mL g^−1^ obtained by the online SEC/DRI 100% mass recovery approach were virtually identical to the erroneous value of (0.168 ± 0.001) mL^−1^ g previously obtained by offline, batch-mode DRI at the same experimental conditions.

The 100% mass recovery method does provide accurate ∂*n*/∂*c* values when using a mix of isorefractive and/or nearly-isovirial solvents. This success, however, is contingent upon the calibration constant of the DRI being accurately determined, the chromatographic peak baseline and integration limits being accurately placed, the concentration of the solution being accurately determined, the analyte being completely dissolved, and all the injected analyte having eluted from the column and being contained within the DRI peak.

The findings here apply not only to size-exclusion chromatography but also to other size-based techniques, such as hydrodynamic chromatography and flow field-flow fractionation methods such as hollow-fiber and asymmetric flow FFF. In these, plate counts are generally lower than in SEC. Hence, the possibility for solvent equilibration in the immediate vicinity of the macromolecule during the course of a chromatographic or fractographic run will also be lower than in SEC.

## Figures and Tables

**Table 1:** ∂*n*/∂*c* values obtained by 100% mass recovery method

Analyte	*M*_w_ (g mol^−1^)	*∂n/∂c* (mL g^−1^)
PS 18K	18,000 ± < 100	0.163
PS 420K	416,000 ± < 1000	0.164
PS 800K	800,000 ± 4000	0.164

*M*_w_ determined by SEC/MALS/DRI in THF; ∂*n*/∂*c* in 25:75 THF:DMF. In both cases, temperature: (25.0 ± 0.1) °C, *λ*_0_ = 658 nm. Results are averages from at least triplicate measurements; standard deviations in ∂*n*/∂*c* are < 0.001 mL g^−1^ in all cases. For details of *M*_w_ determination, see [10]. Note that a ∂*n*/∂*c* value of (0.168 ± 0.001) mL^−1^ g was determined by offline, batch-mode DRI in 25:75 THF:DMF at identical solvent/temperature conditions [10].

## Data Availability

No datasets were generated or analyzed during the current study.
